# Laparoscopic Intraperitoneal Onlay Mesh Plus (IPOM Plus) With Mobilizing the Urinary Bladder for Suprapubic Incisional Hernia Repair: A Case Report

**DOI:** 10.7759/cureus.73117

**Published:** 2024-11-06

**Authors:** Tran Phung Dung Tien, Huan Nguyen Ngoc

**Affiliations:** 1 Digestive Surgery, Cho Ray Hospital, Ho Chi Minh City, VNM

**Keywords:** abdomen ventral hernia, ipom plus, laparoscopic intraperitoneal onlay mesh, mesh repair, suprapubic incisional hernia

## Abstract

Ventral hernias represent a prevalent surgical complication in contemporary medical practice, with incisional hernias being a common long-term outcome following abdominal surgery. There are many risk factors for abdominal incisional hernias, including surgical history, malnutrition, obesity, chronic obstructive pulmonary disease, abdominal closure technique, and surgical site infection. Laparoscopic repair of incisional hernias is the optimal surgical approach, as it is associated with reduced hospital stays, fewer perioperative complications, and lower recurrence rates. This report details a case of suprapubic incisional hernia managed via laparoscopic surgery utilizing adual mesh placed intraperitoneally. The bladder is displaced from the rectus abdominis muscle to form a compartment for the placement of the mesh, which is then anchored to the pubic bone, after which the bladder is sutured back into position. Laparoscopic intraperitoneal onlay mesh plus (IPOM plus) surgery is a feasible alternative to open ventral hernia repair for defects smaller than 10 cm. There is less chance of seroma formation and recurrence with transfascial sutures, which makes them an easy method for defect closure. Using a mesh improves muscle function and reduces the likelihood of hernia recurrence. Recurrence rates are lower and mechanical stability is better with mesh utilization compared to initial suture repair. For us, getting the abdominal wall to approximation and inserting the mesh comfortably require mobilizing the urine bladder.

## Introduction

Ventral hernias are among the most common surgical complications in contemporary medical practice, with incisional hernias being a frequent long-term outcome of abdominal surgery. Incisional ventral hernias that arise within the initial two years after laparotomy or laparoscopic surgery result in significant morbidity; the incidence of these hernias varies from 10% to 20% [1]. Suture materials, surgical techniques, and patient characteristics are just a few of the variables that can affect how incisional hernias develop. Factors influencing surgical operations include incision size, surgical history, body mass index (BMI), obesity, comorbidities such as hypertension and diabetes, chronic obstructive pulmonary disease, and inadequate nutritional condition [2]. Ventral incisional hernias are frequently managed through laparoscopic repair, which offers benefits such as reduced hospital stays, lower wound infection rates, faster healing, and recurrence rates below 5% [3-5]. Laparoscopic ventral hernia repair is increasingly being accepted and adopted globally [6]. The conventional laparoscopic repair of ventral hernias entails securing the defect from the peritoneal aspect with a composite mesh, known as the intraperitoneal onlay mesh (IPOM), and IPOM plus repair entails suturing the fascial defect and reinforcing it with intra-peritoneal mesh [7]. The International Endohernia Society's recommendations are currently this method [8]. Our study details a case of a suprapubic incisional hernia necessitating bladder mobilization to facilitate appropriate mesh insertion. The bladder was then sutured to return it to its natural anatomical place. This work highlights the technical facets of dissection and mesh fixation.

## Case presentation

**Figure 1:**
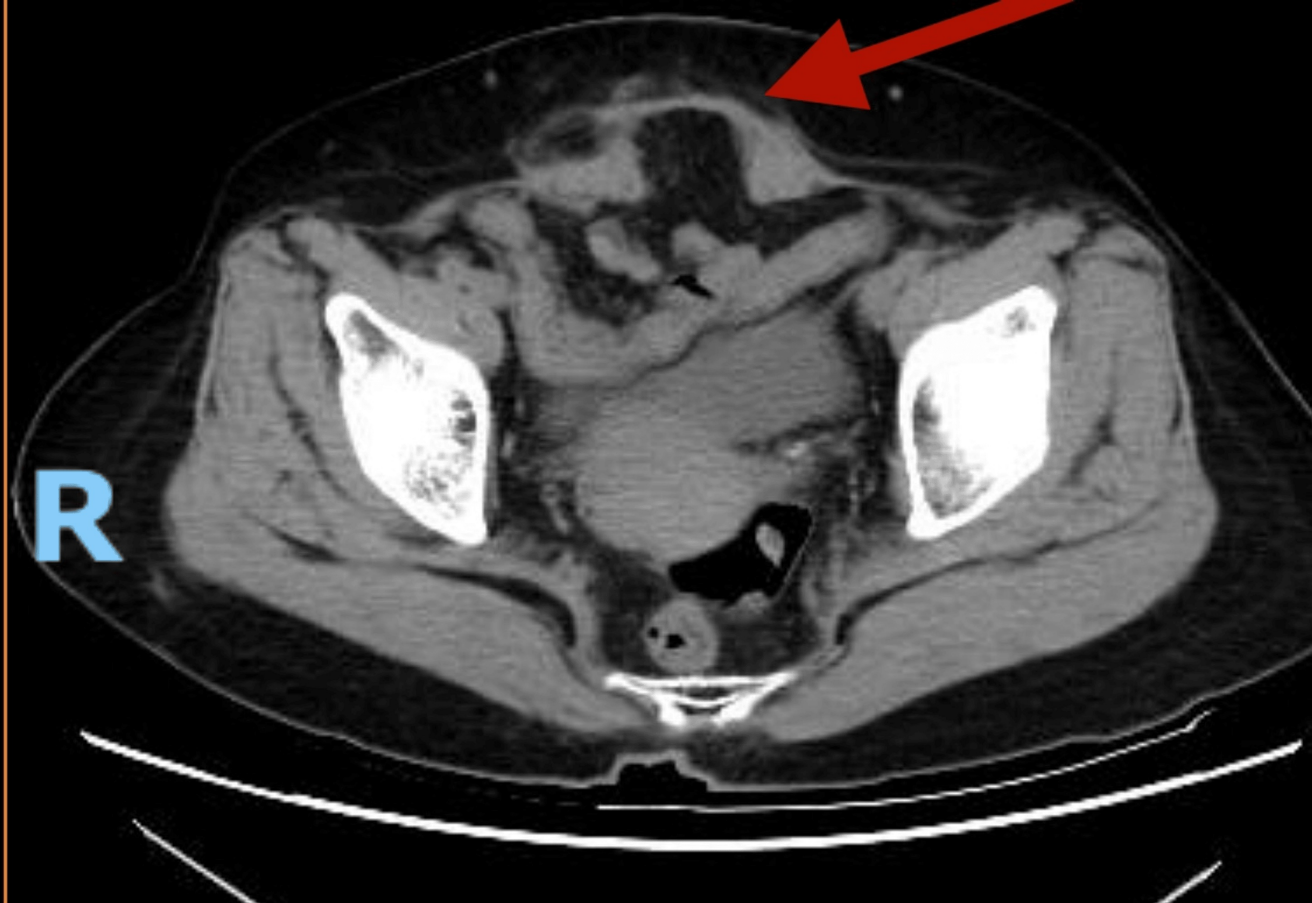
Suprapubic incision hernia (red arrow)

A 74-year-old female patient reported to the digestive surgery department with abdominal pain concentrated near the suprapubic incision. Her medical history included a cesarean section conducted roughly 30 years ago through a transverse suprapubic incision. The patient's body mass index (BMI) was 24 kg/m^2^. The clinical examination of the abdomen identified an incision hernia in the suprapubic scar. A computed tomography (CT) scan verified the existence of a small incisional hernia near the suprapubic scar (Figure 1). A laparoscopic procedure including the placement of a dual mesh intraperitoneally was advised.

Technique

**Figure 2:**
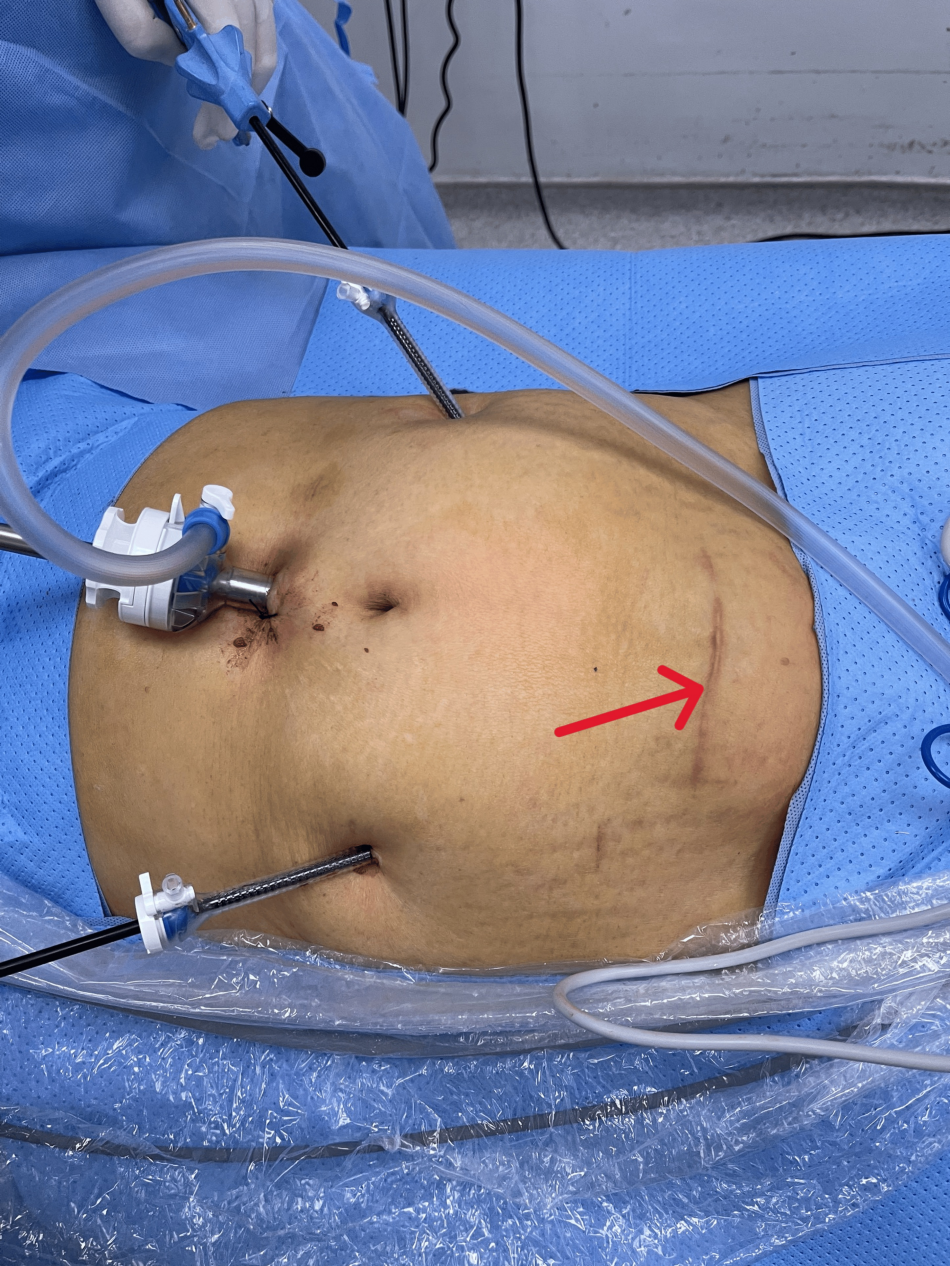
Abdominal Trocar sites and scar (red arrow)

A standard laparoscopic kit and a needle holder were utilized for the surgical procedure. A Foley catheter was inserted prior to the operation. The surgery was conducted laparoscopically, employing three trocars and a camera trocar positioned in the epigastric abdominal cavity, with additional trocars placed bilaterally. The placement of the trocars in the epigastric region facilitated a comprehensive examination of the median abdominal line and the correction of all abnormalities (Figure 2).

**Figure 3:**
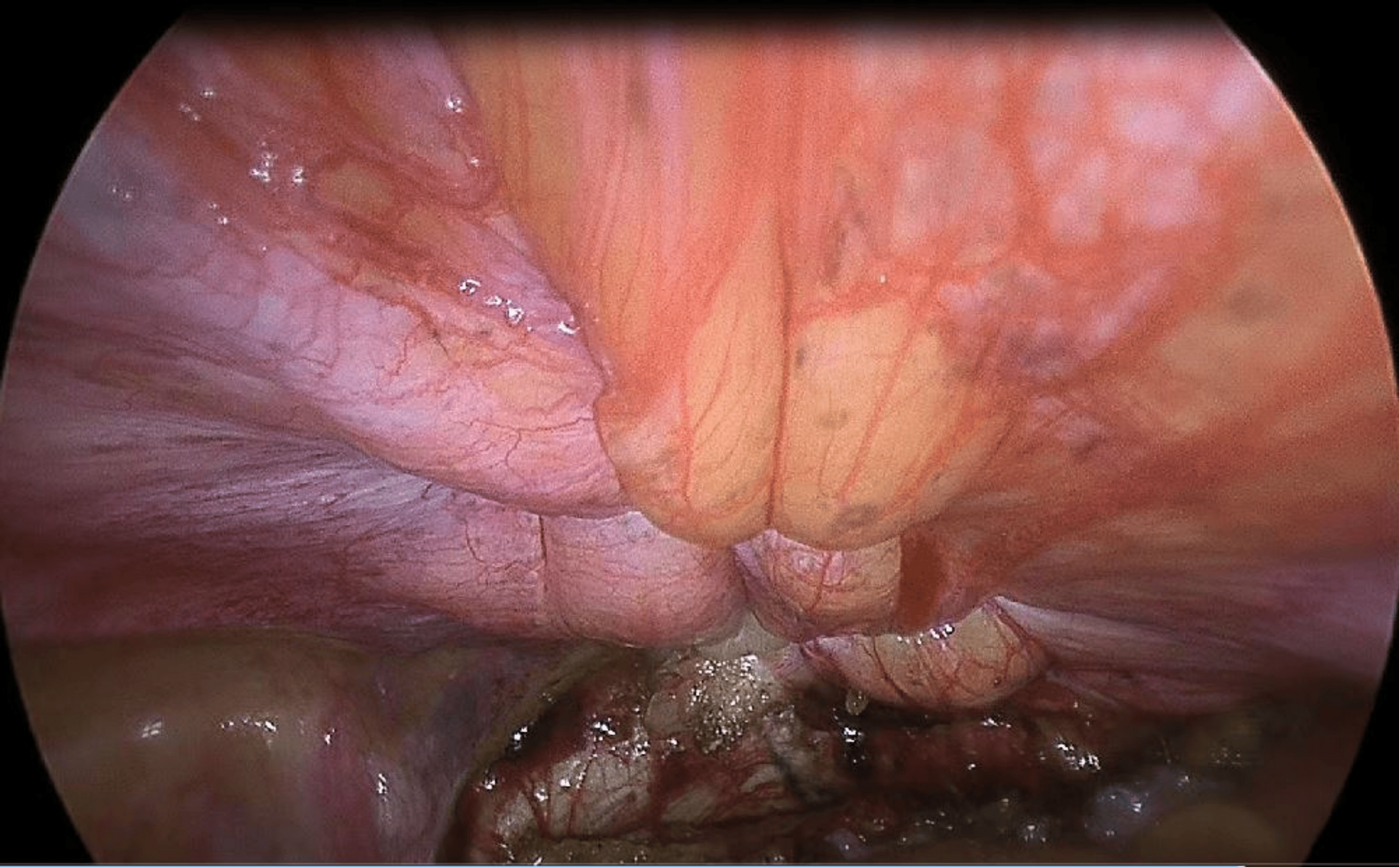
Close facial defect

The evaluation identified an incisional hernia defect on the suprapubic scar, measuring approximately 3.5 x 7 cm. The proximity of the parietal defect to the pubic symphysis necessitated access to the pre-vesical area (Retzius space) to facilitate abdominal wall approximation and mesh insertion. The pre-vesical gap was created by incising the peritoneal layer next to the apex of the urinary bladder. The Retzius plane was accessible, and dissection was performed caudally beneath the pubic bone and laterally to the medial umbilical fold. We close the fascial defect via a few tiny incisions percutaneously using non-absorbable sutures with the needle holder (Prolene 1.0) (Figure 3).

**Figure 4:**
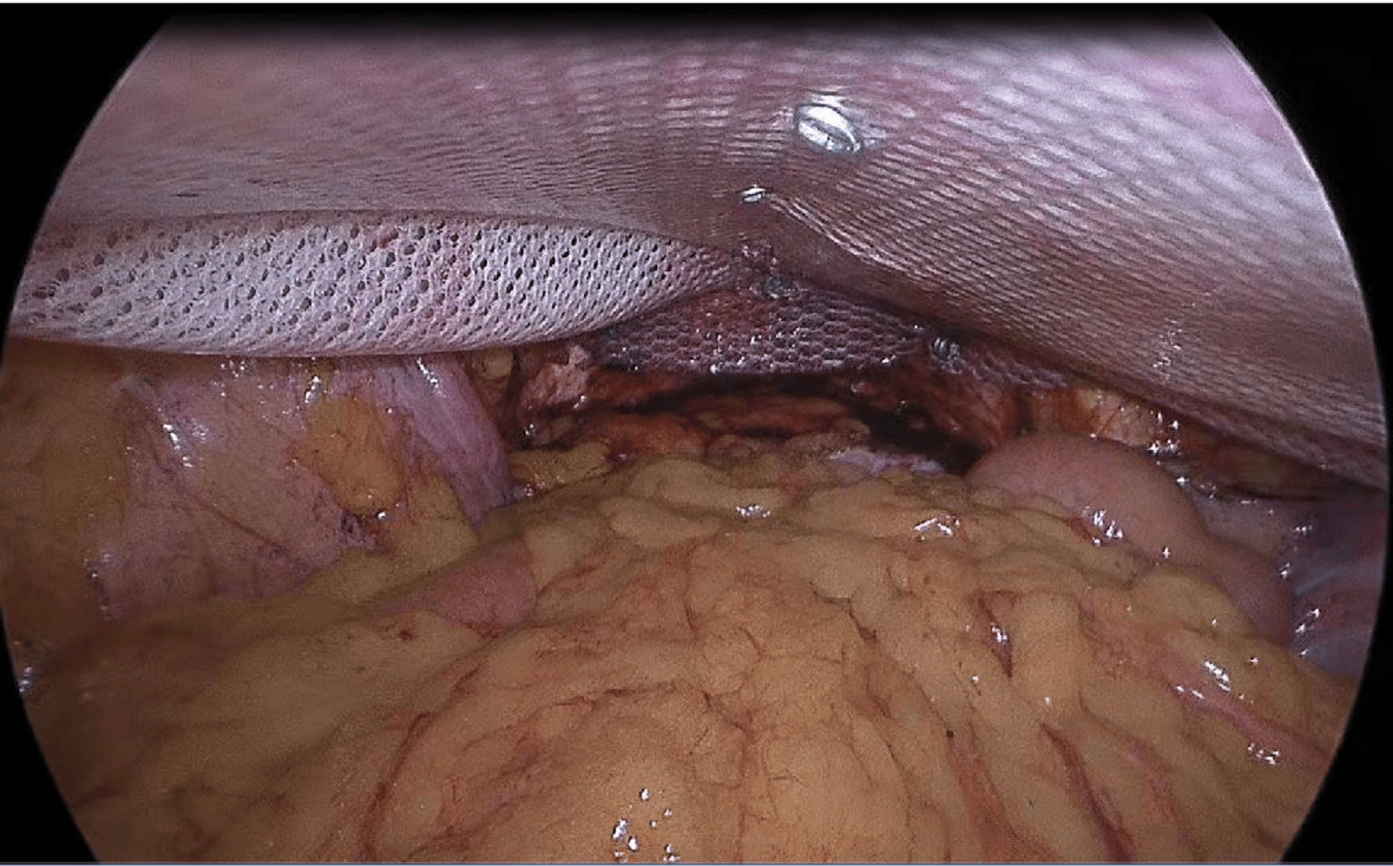
The mesh was affixed to the abdominal wall with non-absorbable tacks

A 20×15 cm dual mesh (Parietex TM Composite Mesh, Covidien, New Haven, CT, USA) was secured to the pubic bone using non-absorbable tacks. The mesh was attached to the abdominal wall using non-absorbable tacks (Figure 4).

**Figure 5:**
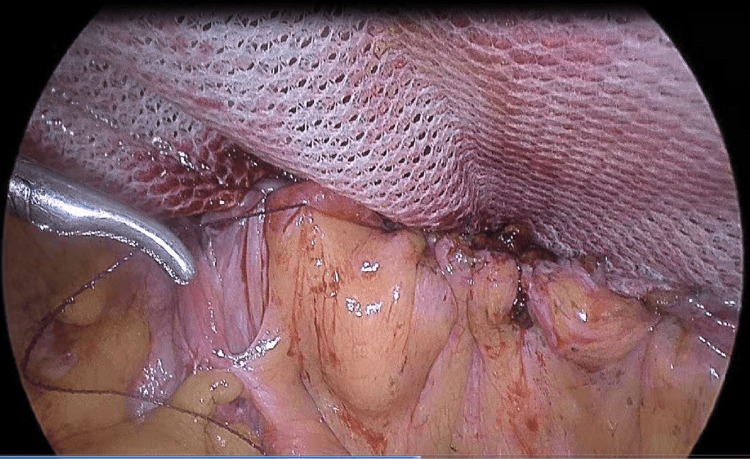
The urinary bladder was reattached to the mesh at its original anatomical location

A continuous absorbable suture (V-loc 3.0 Covidien) was used to attach the urinary bladder back to the mesh in the same position it was in before surgery, lined up with the upper intraperitoneal layer (Figure 5).

The surgical recuperation was uncomplicated. The urinary catheter was removed on the first post-operative day, and the patient spontaneously resumed normal urinating. Two days post-surgery, the patient was discharged with analgesic medicine. At the six-month follow-up, a CT scan showed no hernia recurrences or urinary issues associated with mesh installation.

## Discussion

In 1992, Le Blanc et al. pioneered the initial IPOM laparoscopic procedure, offering a secure alternative to open ventral hernia treatment [6]. The optimal approach for treating incisional hernias via laparotomy or laparoscopy remains undetermined [9,10]. Laparoscopic repair of incisional hernias results in shorter hospital stays, reduced wound infection rates, faster healing, and recurrence rates below 5% [11]. The lifetime risk of developing an incisional hernia in the general population is estimated at 5% [12]. Incisional hernias are a global concern, accounting for 11-20% of all open laparotomy cases. The likelihood of incisional hernia formation is higher in open procedures [13]. Several factors contribute to this, including patient characteristics, the type of primary surgery, and the patient's biological factors [14].

The guidelines advocate for laparoscopic IPOM repair when the defect is less than 10 cm in size. Laparoscopic intervention for larger defects is no longer recommended due to the increased risk of recurrence [15]. Research indicates that addressing defects greater than 10 cm significantly raises the incidence of chronic discomfort necessitating treatment [16]. Suprapubic hernias are predominantly incisional hernias. Repair is difficult because of their proximity to the urinary bladder and reduced fascial support caused by the absence of the posterior rectus sheath beneath the arcuate line. For mesh healing to work best, it should overlap at least 5 cm of the abdominal wall. This spreads the pressure over a larger area and makes it easier for fibrous tissue to grow where the mesh meets the fascia [17].

Lower abdominal hernias necessitate thorough care, encompassing both abdominal wall repair and the mobilization of the urinary bladder to enable mesh implantation. In a study involving 98 patients with ventral hernias, Fan et al. demonstrated that bladder mobilization combined with mesh implantation is both safe and effective [17]. In a systematic review, Köckerling and colleagues determined that the laparoscopic approach to IPOM is associated with fewer post-operative complications, shorter hospital stays, and lower recurrence rates compared to the open technique [12]. The closure of hernia defects with transfascial sutures during laparoscopy is a simple repair technique that minimizes the risk of seroma and recurrence. This minimally invasive method enables mesh insertion and encourages prompt functional recovery of the abdominal wall [18].

Mesh repair demonstrates lower recurrence rates and superior mechanical stability compared to primary suture repair [19]. Nonetheless, possible risks of urinary bladder mobilization encompass bladder damage, urine retention, and hematoma. Kok et al. documented a case of urinary bladder erosion resulting in fistulation to the abdominal wall, which subsequently caused necrotizing fasciitis [20]. In our case, access to the Retzius space was necessary for mesh insertion due to a suprapubic parietal defect. We employed non-absorbable tacks to secure the mesh to the pubic bone, ensuring its stability. Suturing the bladder to the mesh prevents internal hernias and bladder abnormalities.

## Conclusions

Mobilizing the urinary bladder in cases of suprapubic incisional hernia facilitates the proper approximation of the abdominal wall and ensures accurate mesh placement. Laparoscopic surgery for this type of hernia is associated with expedited recovery, reduced risk of infection, and diminished post-operative pain.
